# Deep learning-driven false-lumen volumes predict adverse remodeling better than diameter in patients with residual aortic dissection on CT

**DOI:** 10.1007/s00330-025-12116-9

**Published:** 2025-11-07

**Authors:** Joris Fournel, Mariangela De Masi, Charlotte Lu, Virgile Omnes, Baptiste Muselier, Badih Ghattas, Olivier Bouchot, Moundji Kafi, Alain Lalande, Marine Gaudry, Alexis Jacquier, Axel Bartoli

**Affiliations:** 1 CRMBM—UMR CNRS 7339, Aix-Marseille University, 27, Boulevard Jean Moulin, 13385 Marseille, Cedex 05 France; 2 Visual Computing, DTU Compute, Kongens Lyngby, Denmark; 3 Department of Vascular Surgery, Hôpital de la Timone, AP-HM, Marseille, France; 4 Department of Radiology, Hôpital de la Timone, AP-HM, Marseille, France; 5 Aix-Marseille School of Economics (AMSE), Aix-Marseille University, Marseille, France; 6 Department of Cardio-Vascular and Thoracic Surgery, University Hospital of Dijon, Dijon, France; 7 ICMUB Laboratory, Faculty of Medicine, CNRS UMR 6302, University of Burgundy, Dijon, France; 8 Medical Imaging Department, University Hospital of Dijon, Dijon, France

**Keywords:** Aortic dissection, Computed tomography angiography, Deep-learning, Prognosis, Computer-assisted image processing

## Abstract

**Objectives:**

1. To develop a deep-learning segmentation model for automated measurement of maximal aortic diameter (*D*_max_) and volumes of aortic dissection components: true-lumen (TL), circulating false-lumen (CFL), and thrombus (Th) on CT angiography (CTA). 2. To assess the predictive value of these measures for adverse aortic remodeling in residual aortic dissection (RAD).

**Materials and methods:**

This retrospective study included 322 patients from two centers. The segmentation model was trained on 120 patients (Center 1) and tested on an internal dataset (30 patients, Center 1) and an external dataset (10 patients, Center 2) in terms of Dice Similarity Coefficient (DSC). The model extracted *D*_max_, global false-lumen volume (FL_Glo _= CFL + Th), and local false-lumen volume (FL_Loc_, measured 3 cm around the largest diameter). Clinical validation was performed on 83 patients from Center1 (internal validation, 2-year follow-up) and 79 patients from Center2 (external validation, 4.5-year follow-up).

**Results:**

The segmentation model achieved high accuracy (Center 1, DSC: 0.93 TL, 0.93 CFL, 0.87 Th; Center 2, DSC: 0.92 TL, 0.93 CFL, 0.84 Th) with strong agreement between automated and manual measurements. Aortic remodeling occurred in 39/83 patients (46.9%) from Center1 and 33/79 patients (41.7%) from Center2. Aortic remodeling occurred in 39/83 patients (47%) from Center1 and 33/80 (42%) from Center2. FL_Loc_ outperformed *D*_max_ and FLGlo (Center 1: AUC = 0.83, 0.73, and 0.76; Center 2: AUC = 0.77, 0.64, and 0.70). At optimal thresholds, FL_Loc_ showed good predictive performance (Center 1: Sensitivity = 0.87, Specificity = 0.68).

**Conclusion:**

Deep-learning segmentation provides accurate aortic measurements. Local false-lumen volumes predict adverse aortic remodeling in RAD better than diameter and global false-lumen volumes.

**Key Points:**

***Question*** *In residual aortic dissection (RAD) after type-A dissection, early identification of high-risk patients on initial CT angiography is crucial for endovascular treatment decisions*.

***Findings*** *False-lumen local volumes (3 cm around aortic dissection maximal diameters), obtained with an automatic deep-learning method, predict adverse remodeling better than diameter or global false-lumen volumes*.

***Clinical relevance*** *A deep-learning segmentation method of aortic dissection components on CTA, enabling automatic measurements of diameters and volumes is feasible. It provides local false-lumen volumes, a better predictive marker of adverse aortic remodeling than the currently used diameters and global volumes*.

**Graphical Abstract:**

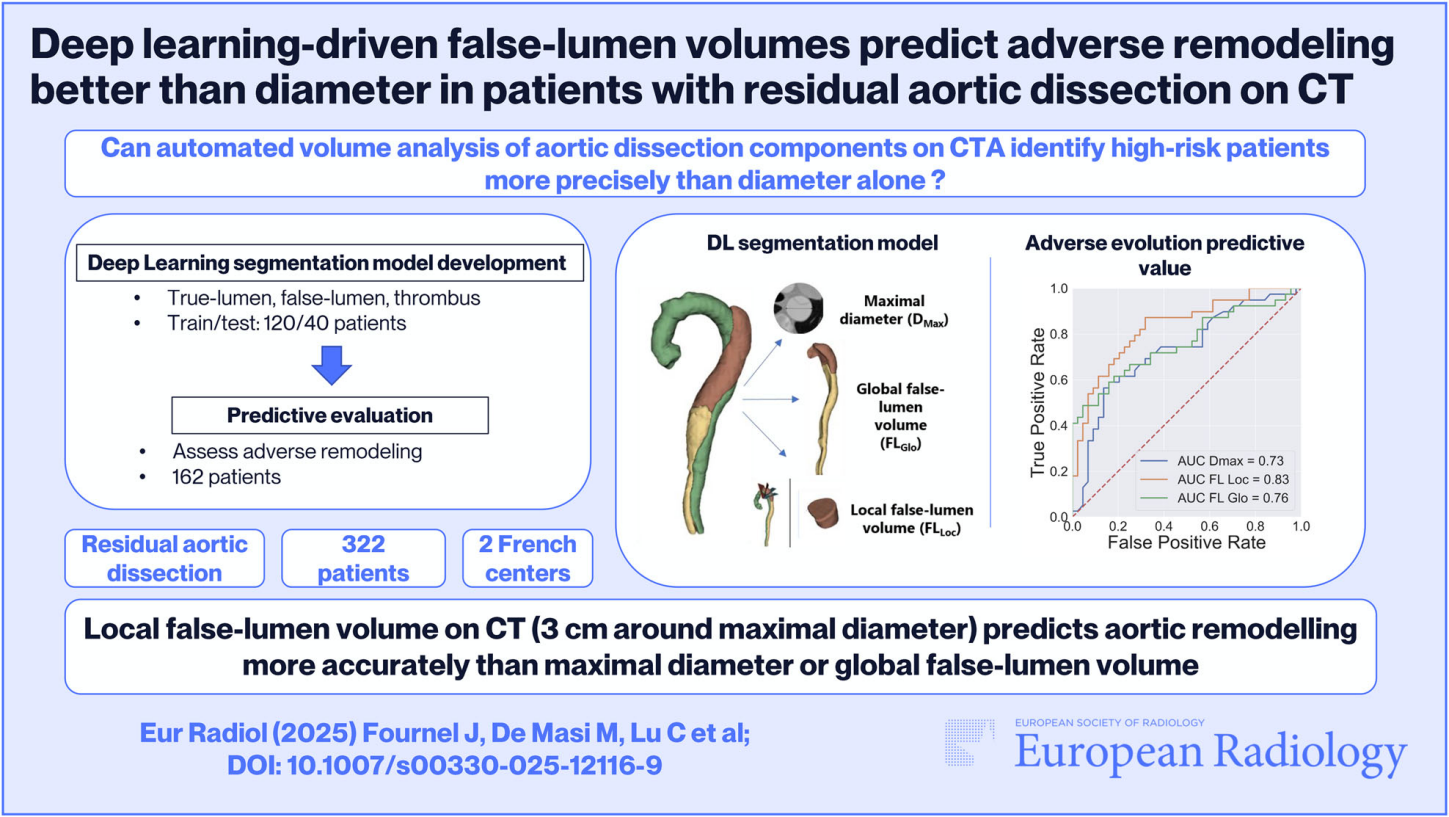

## Introduction

Type A aortic dissection (TAAD) is a life-threatening condition for which the long-term prognosis is associated with the persistence of a residual aortic dissection (RAD) [1]. After acute surgical repair of the ascending aorta, a circulating false lumen (CFL) is a known risk factor for adverse aortic events such as positive remodeling, rupture, malperfusion, and cardiovascular death. Alongside optimal medical treatment, thoracic endovascular aneurysm repair (TEVAR) may be required to occlude the intimal tear and prevent false-lumen growth. The challenge is to identify patients at risk of long-term adverse evolution who could benefit from early TEVAR treatment [2]. The decision is based on imaging findings from computed tomography angiography (CTA) such as maximal aortic diameter (*D*_max_), diameter growth between CTA and entry tear size [3, 4].

However, diameter measurements are mostly carried out manually, entailing a variable degree of reproducibility. Also, they remain a poor predictor of adverse acute aortic events [5]. Recent studies have identified global aortic volume (Ao_Glo_) as a predictor of long-term adverse outcomes [6]. Global aortic volumes assume that all aortic segments are equally important, while dedicated true and false-lumen volumes are more accurate [7]. Also, this method does not assess the presence of a false-lumen thrombus, a known adverse risk marker [8]. There is a need for an automatic segmentation of true-lumen (TL), false-lumen, and thrombus volumes in RAD to assess disease progression and seek out additional new markers predictive of adverse aortic remodeling.

Manual computation of aortic volumes requires time-consuming pixel-level segmentation on CTA, which is incompatible with clinical practice. Several systems using deep-learning (DL) architectures for aortic segmentation have been proposed recently. Current DL methods for aortic segmentation operate either on the raw axial CTA images [9–11] or on multiplanar reconstruction (MPR) images [12, 13]. MPR aortic segmentation yields good results but has limitations: aortic root truncation and significant errors in the diameter measurement of extremely tortuous aortas. In addition, these studies focus on specific groups and do not consider the complete spectrum of aortic dissection (completely thrombosed false-lumen, presence of aortic stent grafts, extreme tortuosity) [14, 15].

Our goals were (1) to develop a fully automated deep learning pipeline that allows segmentation of all aortic dissection components (TL, false-lumen, and thrombus) on CTA. (2) To evaluate the prognostic value of maximal diameter, global and local (around the maximal diameter) volumes of the RAD components in TAAD patients and their evolution over time to predict adverse aortic remodeling.

## Material and methods

### Study design

**Fig. 1:**
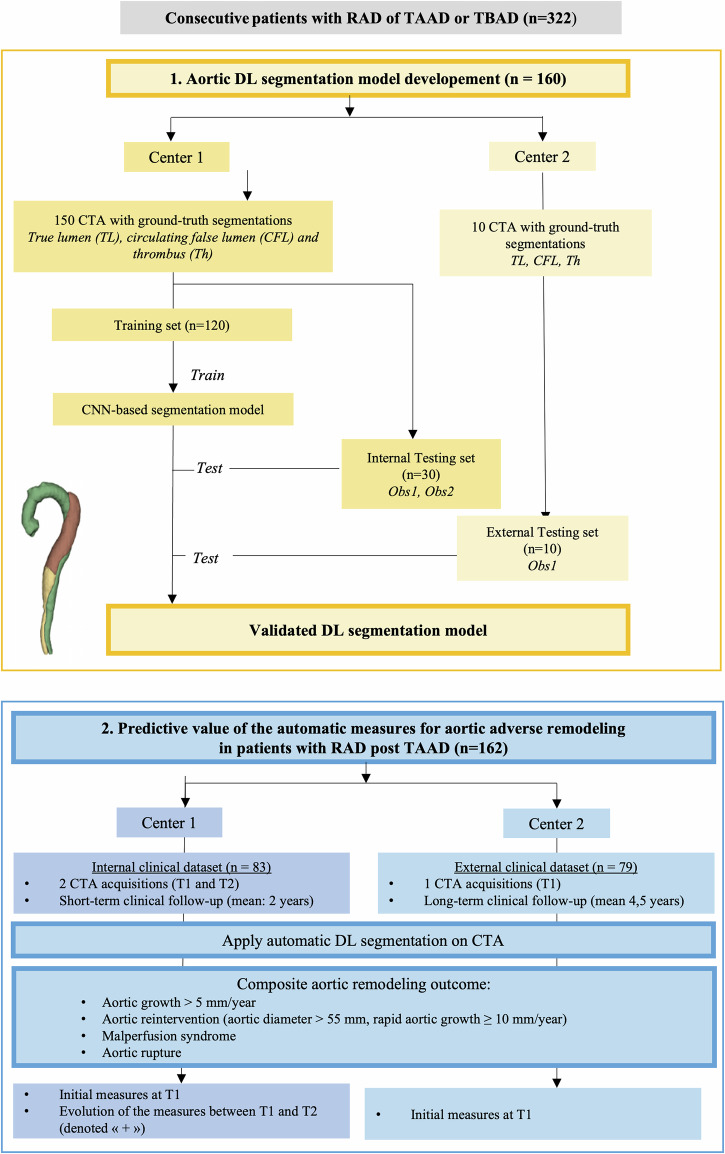
Study design. One block (yellow) for CNN-based segmentation model development and validation. Another block (blue) for assessing the predictive value of automatically obtained measures on clinical datasets. RAD, residual aortic dissection; TAAD, type A aortic dissection; TBAD, type B aortic dissection; CTA, computed tomography angiography; CNN, convolutional neural network

This double-center retrospective, observational study was conducted from January 2017 to December 2023 in La Timone Hospital (Assistance-Publique/Hôpitaux de Marseille, France (Center 1) and in Dijon University Hospital (Dijon, France) (Center 2) and followed the RGPD rules as well as the MR-004 rules (IRB 2019-48 and Health Data Hub 22683280). This study was divided into two parts conducted on different datasets (1) to develop a DL pipeline that enabled us to obtain automatic aortic dissection components: segmentation, diameters and volumes (2) to assess the predictive value of automatically obtained measurements to predict the adverse evolution of RAD on an internal validation dataset (from Center 1) and an external validation with longer clinical follow-up (Center 2). A flow chart is presented in Fig. 1.

### Population and data

All patients referred to both institutions for TAAD with RAD after surgical repair of the ascending aorta or with type B aortic dissection (TBAD) and a total aortic CTA were included. Exclusion criteria were patients with TAAD before aortic surgery, incomplete aortic CTA, or significant artifacts.

The DL model was trained and tested on a segmentation dataset from Center 1. The segmentation dataset patients had one CTA and were randomly split into a training and a testing set. Additionally, 10 patients from Center 2 were randomly selected to conduct an external testing validation.

Secondly, the clinical validation of the model’s predictive value was conducted on distinct populations from both centers, named clinical datasets: one from Center 1 for internal clinical validation and one from Center 2 for external clinical validation. For this subset of patients, the inclusion criteria were more restrictive and included only patients who had a RAD post-TAAD with clinical follow-up for at least 2 years. For Center 1 patients, radiological follow-up for each patient included a first aortic CTA (T1) performed immediately after TAAD surgery and a second aortic CTA (T2) performed within one year post-T1. All patients from the Center 1 clinical dataset have been reported in a previous paper [16]. For Center 2 patients, only the first postoperative CTA (T1) was available.

Patients from the segmentation datasets were excluded from the clinical datasets. While the clinical dataset focused on RAD, the training dataset included both RAD and TBAD to increase the training sample size, as they share phenotypic similarities in key segmentation features.

Aortic CTA examination acquisitions from both centers are presented in Supplemental Material A.

### Development and evaluation of a DL aortic segmentation pipeline

#### Manual segmentation and aortic measurements

**Fig. 2:**
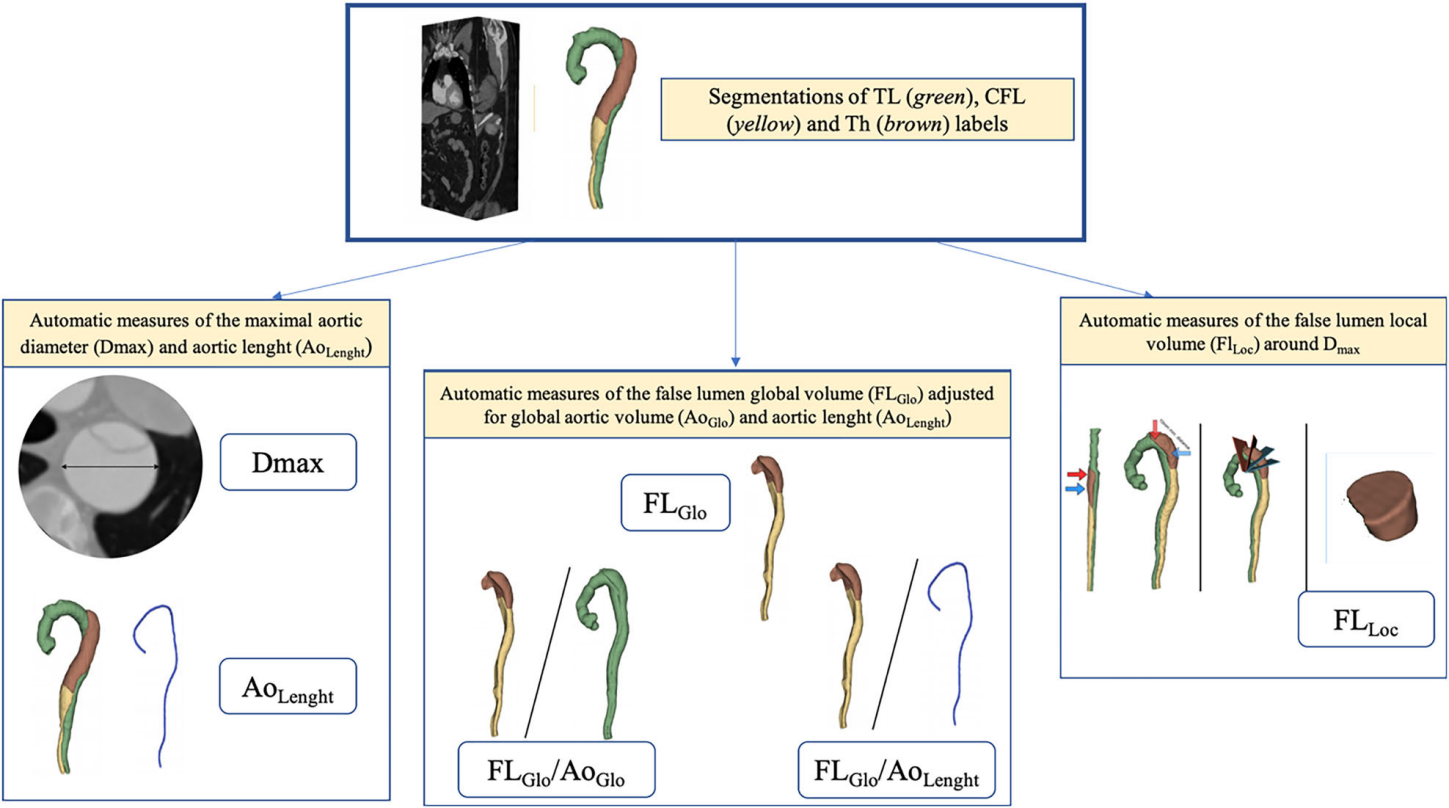
Representation of the aortic label segmentations and the different aortic measures automatically derived from the segmentations. Tl, true-lumen; CFL, circulating false-lumen; TH, thrombus; *D*_max_, maximal aortic diameter; Ao_Length_, aortic length; FL_Glo_, global false-lumen volume; FL_Glo_ /Ao_Glo_, global false-lumen over global aortic volume; FL_Glo_/Ao_Length_, global false-lumen volume over vessel length; FL_Loc_, local false-lumen volume

The same radiologist carried out manual image segmentations. (C.L.,7 years experience; Obs1) using 3DSlicer [17] on the segmentation dataset. A second observer (A.B., 9 years of experience; Obs2) manually segmented the 30 CTA from the internal testing dataset, blinded from Obs1 results. CTA DICOM images were extracted anonymously, and the aorta was segmented from the aortic valve to the aorto-iliac bifurcation into the TL, the CFL, and the thrombus (TH) if present, excluding the aortic branches but including the aortic wall. If the arterial acquisition was unable to distinguish FL from TH, a delayed phase was used. The manual segmentations were considered as the reference standard to train a segmentation model and evaluate its performance. Model development details are given in Supplemental Material B. Aortic measurements were obtained from the segmentations. The maximum aortic diameter (*D*_max_, mm), was obtained from double-oblique segmentations, and aortic length (Ao_length_, mm), from the aortic valve to the aorto-iliac bifurcation. Volumes of TL, CFL, and Th (mL) were obtained, and their sum was the global aortic volume (Ao_Glo_) [6]. Global false-lumen (FL_Glo_) was the sum of CFL + Th. FL_Glo_ adjusted to the Ao_length_ and Ao_Glo_. Finally, local false-lumen volume (FL_Loc_) was calculated. This consists of locally restricting the false-lumen volume measurement to a 30-mm section (15 mm proximal to 15 mm distal) local section around a large diameter aortic position. This measurement is repeated for each of the two largest diameters, spaced by at least 10 mm, and the highest value of the two local false lumen volumes is retained. All measurements are presented in Fig. 2.

### Predictive value of automatic aortic measurements

**Fig. 3:**
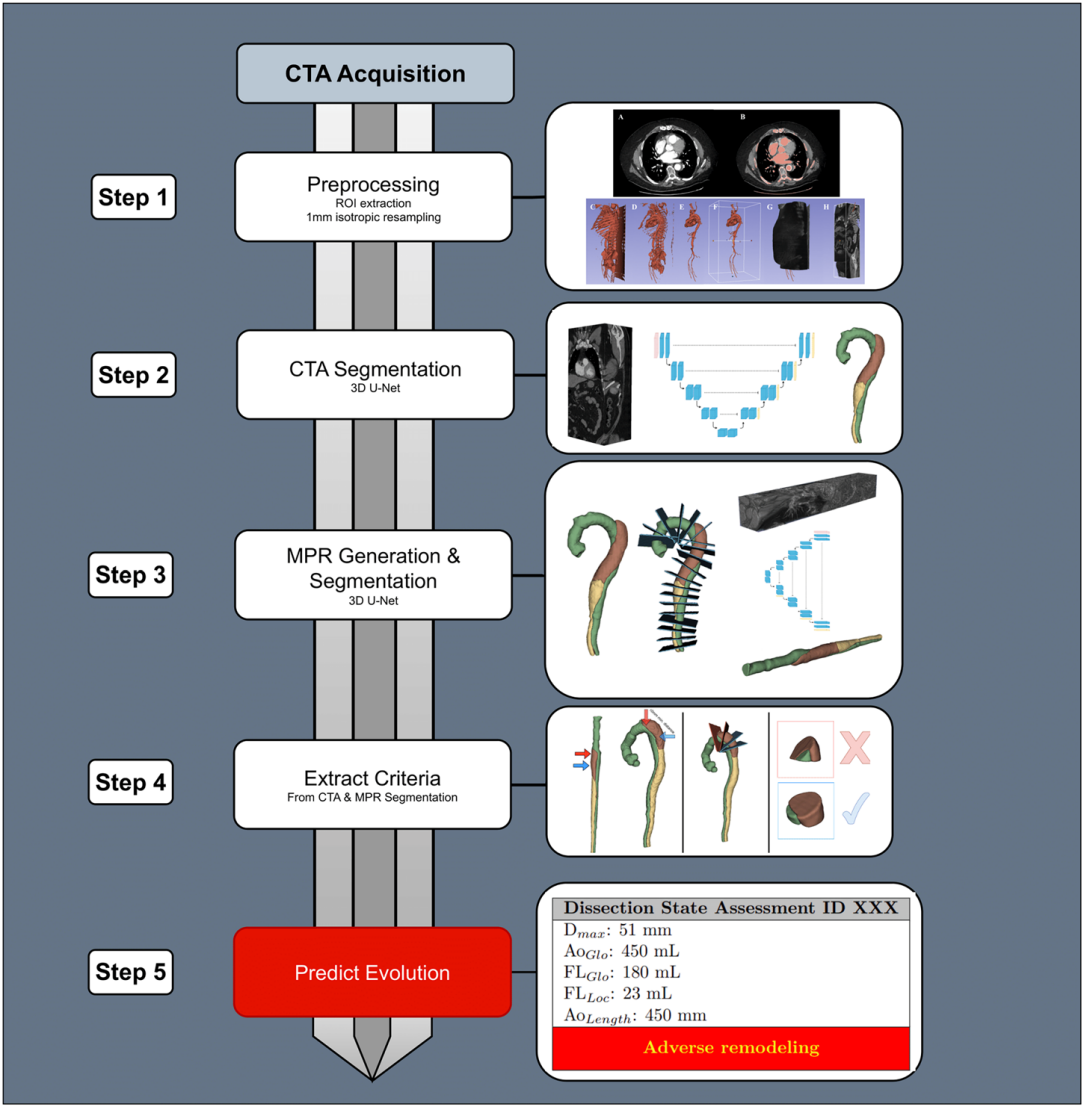
Model description in five steps. CTA, computed tomography angiography; ROI, region of interest; MPR multiplanar reconstruction; *D*_max,_ maximal aortic diameter; Ao_Glo_, global aortic volume_;_ Ao_Length_, total aortic length; FL_Glo_, global false-lumen volume; FL_Loc_, local false-lumen volume

The second part of the study was to assess the predictive value of the automatically obtained aortic measurements on the clinical datasets. Images from T1 (Centers 1 and 2) and T2 CTA (Center 1) were automatically segmented through the DL model (Fig. 3), and aortic measures were obtained. In addition to the baseline morphological criteria at T1, the evolution of these measurements between T1 and T2 was computed. To account for the variability in the number of days between T1 and T2, the difference in days was standardized using time coefficient multiplication before adding this difference to the initial T1 measure (Supplementary Material C.1). The resulting measurement represents an estimation of the measurement one year after T1. Those evolution time-normalized measurements at T2 are denoted “+”: for example, *D*_max_ (T1) and *D*_max_ + (time-normalized evolution at T2). The endpoint was adverse aortic remodeling during follow-up, defined by one of the following criteria: aortic growth > 5 mm/year or aortic re-do surgery (indicated for aortic diameter > 55 mm, rapid aortic growth ≥ 10 mm/year) or malperfusion syndrome or aortic rupture.

### Statistical analysis (J.F.)

All results were expressed as mean ± standard deviation. *p*-values under 0.05 were considered significant. A paired Student’s *t*-test was used to assess the significance of bias when the sample size was ≥ 30 or when a Shapiro test showed that the sample was normal. Wilcoxon testing was used for the DSC. To assess the predictive value of the different measurement areas under the ROC curve (AUC) with a 95% confidence interval (95% CI), likelihood-ratio tests were also used. Kolmogorov-Smirnov statistics were used to measure the distance between distributions. All statistical analyses were performed using Python and the Spicy Stats library.

## Results

### Population characteristics

Three hundred and twenty-two patients were included in the analysis. The segmentation dataset was composed of 160 patients, 120 for training, 40 to test the model (30 from Center 1, and 10 from Center 2). Segmentation dataset characteristics are presented in Table 1. Mean *D*_max_ in the training dataset was 52.3 mm ± 9.0, 49.5 mm ± 5.2 in the internal testing dataset, and 51.0 ± 13.3 in the external testing dataset. In the training dataset, sixty patients (50%) had a partially or totally thrombosed FL, twenty-one (16.7%) had an aortic stent-graft, and sixteen (13.3%) had extreme tortuosity, defined by a maximum tortuosity angle greater than 45 degrees [18]. FL_Glo_ was higher in the external testing set than in the internal testing set (305.3 ± 281.6 vs. 208.0 ± 101.8, *p* < 0.001). The clinical datasets for both Center 1 and Center 2 are presented in Table 2. In Center 1, 39 patients presented with adverse aortic remodeling during follow-up, with 23 (59.0%) cases of aortic growth, 14 (35.9%) aortic re-do surgery, 2 (5.1%) with malperfusion syndrome, and 0 (0%) with aortic rupture. In Center 2, 33 (41.7%) patients had adverse aortic outcomes, with one notable aortic rupture (3%).

**Table 1:** Baseline characteristics of the segmentation dataset

	Segmentation dataset (*n* = 160)		
	Training dataset (*n* = 120)	Testing dataset (*n* = 40)	
	Center 1	Internal—Center 1	External—Center 2
Population, *n*	120	30	10
Male, *n* (%)	90 (75.0)	20 (66.6)	7 (70.0)
Age, yo, mean ± SD	58.8 ± 15.4	55.0 ± 16.0	65.6 ± 9.8
RAD of TAAD, *n* (%)	35 (29.2)	12 (40)	10 (100)
TBAD,* n* (%)	85 (70.8)	18 (60)	0 (0)
Specific aortic dissection variants			
Partially thrombosed false-lumen, *n* (%)	60 (50)	15 (50)	3 (33.3)
Aortic metallic stent-graft, *n* (%)	21 (16.7)	7 (23.3)	0 (0)
Extreme tortuosity, *n* (%)	16 (13.3)	5 (16.7)	3 (33.3)
Morphologic aortic measures			
* D*_max_, mm, mean ± SD	52.3 ± 9.0	49.5 ± 5.2	51.0 ± 13.3
Ao_Glo_, mL, mean ± SD	495.6 ± 173.9	441.8 ± 93.4	519.6 ± 268.5
TL, mL, mean ± SD	245.5 ± 109.8	233.5 ± 104.2	214.3 ± 80.3
CFL, mL, mean ± SD	180.5 ± 124.8	170.9 ± 108.4	251.6 ± 155.9
Th, mL, mean ± SD	69.6 ± 103.0	37.9 ± 33.8	53.7 ± 125.7
FL_Glo_, mL, mean ± SD	250.1 ± 130.2	208.0 ± 101.8	305.3 ± 281.6
FL_Loc_, mL, mean ± SD	36.6 ± 28.0	27.5 ± 20.0	39.8 ± 25.1
FL_Glo_ /Ao_Glo_, mean ± SD	0.49 ± 0.16	0.47 ± 0.18	0.56 ± 0.08
FL_Glo_ /Ao_Length_, mL/mm, mean ± SD	0.53 ± 0.28	0.43 ± 0.21	0.58 ± 0.33

*SD* standard deviation, *RAD* residual aortic dissection, *TAAD* type A aortic dissection, *TBAD* type B aortic dissection, *D*_max_ maximal aortic diameter, *Ao*_Glo_ global aortic volume, *TL* true-lumen, *CFL* circulating false-lumen, *Th* thrombus, *FL*_Glo_ global false-lumen volume, *FL*_Loc_ local false-lumen volume, *Ao*_Lengthh_ total aortic length

**Table 2:** Baseline characteristics of the clinical datasets

	Center 1 (*n* = 83)	Center 2 (*n* = 79)
Male, *n* (%)	63 (75.9)	61 (77.2)
Age, yo, mean (± SD)	61.5 (± 10.9)	62.0 (± 9.7)
Hypertension, *n* (%)	49 (59.0)	50 (63.2)
Hyperlipidemia, *n* (%)	14 (16.9)	19 (24.0)
Smoking, *n* (%)	17 (20.4)	7 (8.8)
Diabetes, *n* (%)	2 (2.4)	4 (5.0)
COPD, *n* (%)	7 (8.4)	4 (5.0)
Marfan syndrome, *n* (%)	9 (10.8)	2 (2.5)
RAD post TAAD	83 (100)	79 (100)
Adverse aortic remodeling, *n* (%)	39 (46.9)	33 (41.7)
Aortic growth, *n* (%)	23 (59.0)	5 (15.1)
Aortic reintervention, *n* (%)	14 (35.9)	18 (53)
Aortic rupture, *n* (%)	0 (0)	1 (3)
Malperfusion syndrome, *n* (%)	2 (5.1)	9 (27.3)

*SD* standard deviation, *COPD* chronic obstructive pulmonary disease, *RAD* residual aortic dissection, *TAAD* *type A* aortic dissection

### Segmentation model performance

**Fig. 4:**
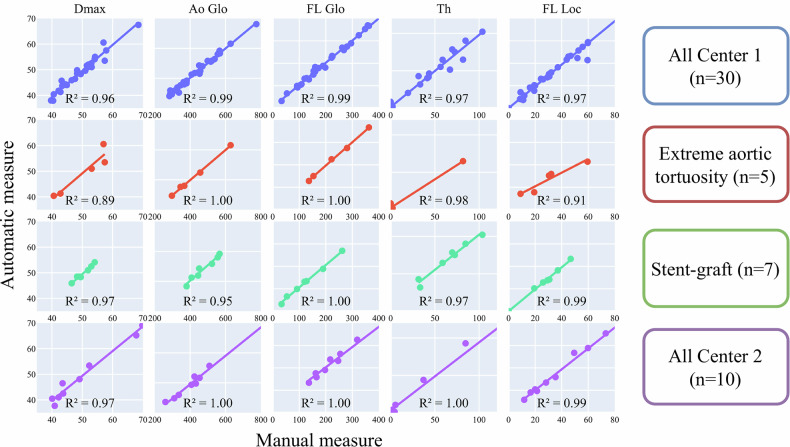
Correlation analysis of the different measurements between automated and manual measures on the internal testing dataset (center 1, *n* = 30) and external testing dataset (center 2, *n* = 10). *D*_max,_ maximal aortic diameter; Ao_Glo_, global aortic volume; FL_Glo_, global false-lumen volume; Th, thrombus; FL_Loc_, local false-lumen volume. Colored lines represent the fitted regression line. Tortuous represents patients of the internal testing dataset with extreme aortic tortuosity (*n* = 5). Stent-graft represents patients of the internal testing dataset with aortic stent-graft (*n* = 7)

The automated model performances are presented in Table 3. The automated segmentation model achieved high DSC scores across all classes for the internal testing dataset (AoGlo: 0.95 ± 0.02, TL: 0.93 ± 0.02, CFL: 0.93 ± 0.04, Th: 0.87 ± 0.06) and for the external testing dataset (AoGlo: 0.94 ± 0.02, TL: 0.92 ± 0.02, CFL: 0.93 ± 0.02, Th: 0.84 ± 0.08) and had moderate mean absolute errors with no significant bias. On the internal dataset, it outperformed Obs2 for AoGlo and TL DSC (*p* = 0.03 and *p* = 0.02, respectively) and showed a significantly lower TL volume MAE (6.7 ± 6.0 mL vs. 10.0 ± 8.3 mL, *p* = 0.040). The segmentation time was drastically reduced (5 ± 2 min vs. 35 ± 10 min manually). Strong correlations with manual reference segmentation were observed across all metrics for both internal and external testing (Fig. 4). *D*_max_ and FL_Loc_ measures correlation were high in the internal dataset (*R*² = 0.96 and *R*² = 0.97, respectively) and in the external dataset (*R*² = 0.97 and *R*² = 0.99, respectively), with high consistency even in challenging cases (e.g., aortic stent-grafts, *R*² = 0.97 and *R*² = 0.99, respectively).

**Table 3:** Model segmentation performance on the testing dataset (*n* = 40)

	Internal—Center 1			External—Center 2	
	Model vs. Obs1	Obs2 vs. Obs1	*p*^†^	Model vs. Obs1	*p*^†^
**Segmentation labels**					
True-lumen, TL					
DSC	0.93 ± 0.02	0.92 ± 0.03	**0.02**	0.92 ± 0.02	**0.02**
MAE, mL	6.7 ± 6.0	10.0 ± 8.3	**0.04**	11.9 ± 9.0	**0.04**
Bias, mL (p*)	0.02 ± 9.1 (0.99)	−3.1 ± 12.8 (0.19)		−7.9 ± 12.9 (0.09)	
Circulating false-lumen, CFL					
DSC	0.93 ± 0.04	0.93 ± 0.03	0.67	0.93 ± 0.02	0.39
MAE, mL	6.4 ± 6.0	6.6 ± 4.7	0.88	11.6 ± 12.6	0.10
Bias, mL (p*)	1.8 ± 8.6 (0.27)	0.9 ± 8.1 (0.52)		−5.5 ± 16.6 (0.32)	
Thrombus, Th					
DSC	0.87 ± 0.06	0.85 ± 0.09	0.57	0.84 ± 0.08	0.66
MAE, mL	5.2 ± 5.5	5.0 ± 5.2	0.90	10.2 ± 13.0	**0.03**
Bias, mL (p*)	−0.1 ± 7.7 (0.97)	−0.1 ± 7.3 (0.95)		−2.9 ± 16.9 (0.68)	
Global aortic volume, Ao_Glo_					
DSC	0.95 ± 0.02	0.94 ± 0.02	**0.03**	0.94 ± 0.01	0.21
MAE, mL	11.0 ± 8.9	10.6 ± 9.5	0.73	21.9 ± 14.1	**0.02**
Bias, mL (p*)	2.2 ± 14.1 (0.42)	−2.3 ± 14.2 (0.37)		−15.0 ± 21.9 (0.06)	
**Morphologic measures**					
Maximal diameter, *D*_max_					
MAE, mm	1.1 ± 1.0	1.3 ± 1.2	0.47	1.6 ± 1.0	0.20
Bias, mm (p*)	−0.5 ± 1.0 (0.07)	−0.1 ± 1.8 (0.76)		−0.6 ± 1.9 (0.36)	
False-lumen local volume, FL_Loc_					
MAE, mL	2.1 ± 3.4	1.7 ± 1.9	0.46	2.5 ± 2.1	0.72
Bias, mL (p*)	0.9 ± 3.9 (0.22)	0.8 ± 2.4 (0.10)		−1.3 ± 3.1 (0.23)	

For all metrics, the mean ± standard deviation of the values is reported

*p*^†^ represents the difference between model performance and inter-observer performance (Wilcoxon) in Center 1 and the difference (Mann–Whitney) between Center 1 model performance and Center 2 model performance. Significant mean differences between model and Obs2 measures are given in bold print

p* represents the significance of the bias (*t*-test)

*DSC* dice similarity coefficient, *MAE* mean absolute error, *TL* true-lumen, *CFL* circulating false-lumen, *Th* thrombus, *Ao*_*Glo*_ global aortic volume, *D*_max_ maximal aortic diameter, *FL*_Glo_ global false-lumen volume, *FL*_Loc_ local false-lumen volume

### Predictive value of automatic aortic measures on RAD patients

The Center 1 clinical dataset was composed of 83 patients who had a RAD post TAAD with clinical follow-up of 2 years and radiological follow-up (Table 4). The mean time between T1 and T2 was 194 ± 77 days. Six patients had incomplete aortic arch exploration on T2 CTA and were excluded from evolution analysis at T2, but kept for T1-based analysis. The Center 2 clinical dataset was composed of 79 patients with a mean clinical follow-up of 4.5 years, with only one T1 CTA.

**Table 4.** Morphologic and evolutionary measures for the clinical datasets from both centers

		Center 1				
	**Overall** **dataset at T1**	**Adverse remodeling events**		**No adverse aortic remodeling events**		
	**(*n*** = **83)**	**T1 (*n*** = **39)**	**T2 (*n*** = **37)**	**T1 (*n*** = **44)**	p*	**T2 (*n ***= **40)**
Morphologic measures						
* D*_max_, mm	41.9 ± 5.1	44.0 ± 5.1	47.8 ± 6.7	40.0 ± 4.5	**< 0.001**	41.4 ± 5.0
Ao_Glo_, mL	345.5 ± 98.7	382.2 ± 118.6	426.4 ± 156.7	312.9 ± 61.8	**0.001**	321 ± 75.2
TL, mL	199.9 ± 71.0	197.3 ± 81.2	203.7 ± 93.1	202.3 ± 61.5	0.75	211.5 ± 72.9
CFL, mL	129.4 ± 80.9	163.8 ± 86.3	178.5 ± 113.7	98.9 ± 62.2	**< 0.001**	96.9 ± 71.3
Th, mL	16.2 ± 28.6	21.1 ± 34.8	44.2 ± 70.2	11.8 ± 21.2	0.13	12.7 ± 24.5
FL_Glo_, mL	145.6 ± 81.7	185.0 ± 87.9	222.8 ± 130.3	110.7 ± 56.9	**< 0.001**	109.6 ± 73.5
FL_Loc_, mL	15.8 ± 10.9	22.4 ± 9.9	30.1 ± 15.8	10.0 ± 8.1	**< 0.001**	11.5 ± 9.6
FL_Glo_/Ao_Glo_, mL/mL	0.41 ± 0.2	0.47 ± 0.2	0.50 ± 0.2	0.35 ± 0.2	**< 0.001**	0.34 ± 0.2
FL_Glo_ /Ao_Length_, mL/mm	0.35 ± 0.2	0.44 ± 0.2	0.53 ± 0.3	0.27 ± 0.1	**< 0.001**	0.27 ± 0.2
Evolution measures						
*D*_max_ + , mm	51.4 ± 13.7	x	57.1 ± 14.3	x	X	46.1 ± 10.9
FL_Glo_ + , mL	249.6 ± 236.1	x	373.2 ± 277.3	x	X	138.0 ± 105.8
FL_Loc_ + , mL	77.0 ± 62.3	x	98.2 ± 72.9	x	X	29.2 ± 29.5
		**Center 2**				
	Overall dataset at T1	**Adverse remodeling events**		**No adverse aortic remodeling events**		
	**(*n*** = **79)**	**T1 (*n*** = **33)**		**T1 (*n*** = **46)**		
Morphologic measures						
*D*_max_, mm	42.0 ± 6.0	43.3 ± 6.4		40.9 ± 5.6	**< 0.001**	
Ao_Glo_, mL	359.5 ± 88.0	390.0 ± 87.6		334.6 ± 81.0	**0.004**	
TL, mL	193.5 ± 59.5	196.4 ± 67.5		191.1 ± 52.7	0.70	
CFL, mL	144.6 ± 77.6	166.6 ± 72.1		126.6 ± 78.0	**0.02**	
Th, mL	21.4 ± 36.4	27.0 ± 45.0		16.8 ± 27.1	0.21	
FL_Glo_, mL	166.1 ± 76.3	193.6 ± 70.8		143.5 ± 73.8	**0.002**	
FL_Loc_, mL	17.8 ± 9.1	22.4 ± 8.8		14.3 ± 7.6	< **0.001**	
FL_Glo_/Ao_Glo_, mL/mL	0.45 ± 0.15 0.34 ± 0.15	0.50 ± 0.13 0.40 ± 0.15		0.41 ± 0.16 0.29 ± 0.13	**0.016** **0.001**	
FL_Glo_ /Ao_Length_, mL/mm						

All results are presented in mean (± SD). p* represents the significance of the mean difference (*t*-test) between groups at T1.

Significant differences are given in bold print

*D*_max,_ maximal aortic diameter; *Ao*_Glo_, global aortic volume_;_ *TL*, true-lumen; *CFL*, circulating false-lumen; *Th*, thrombus; *FL*_Glo_, global false-lumen volume; *FL*_Loc_, local false-lumen volume; *Ao*_Length_, total aortic length

Mean *D*_max_ at T1 was significantly higher for patients with adverse aortic events than patients without in Center 1 (44.0 mm ± 5.1 vs. 40.0 mm ± 4.5, *p* < 0.001) and Center 2 (43.3.0 mm ± 6.4. vs. 40.9 mm ± 5.6, *p* < 0.001). FL_Loc_ at T1 was higher in the adverse events group in Center 1 (22.4 mL ± 9.9 vs. 10.0 mL ± 8.1, *p* < 0.001) and in Center 2 (22.4 mL ± 8.8 vs. 14.3 mL ± 7.6, *p* < 0.001). FL_Glo_/Ao_Length_ ratio was significantly higher at T1 for patients with the outcome than with patients without (0.44 ± 0.2 vs. 0.27 ± 0.1, respectively, *p* < 0.001).

**Fig. 5:**
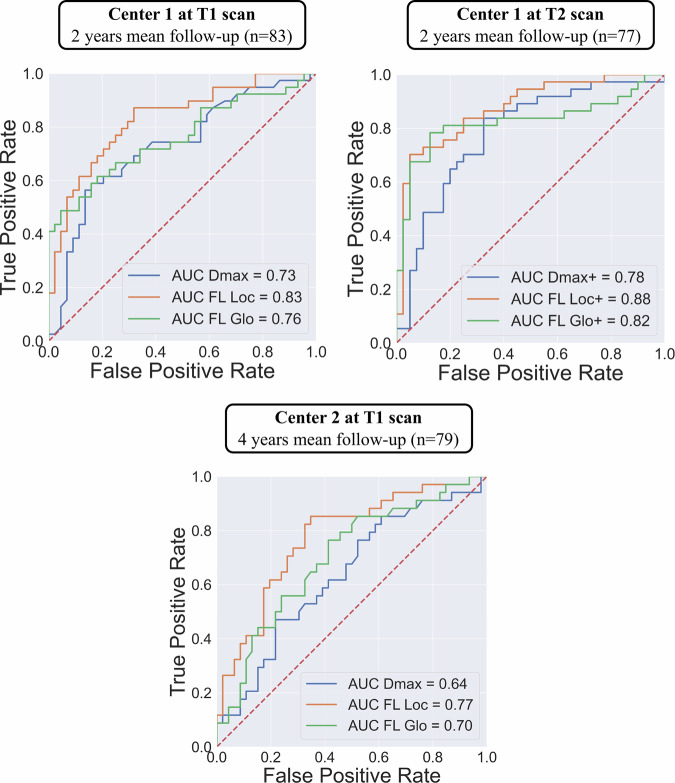
ROC curves AUC for morphologic and evolution measures. AUC, area under curve; *D*_max,_ maximal aortic diameter; *FL*_Glo_, global false-lumen volume; *FL*_Loc_, local false-lumen volume

ROC curves are presented in Fig. 5. FL_Loc_ was a stronger predictor of adverse aortic remodeling than *D*_max_ and FL_Glo_ with AUC of 0.83, 0.73, and 0.76, respectively, for Center 1 and 0.77, 0.64, and 0.70, respectively, for Center 2. Concerning evolution markers with time-adjusted values between T1 and T2 in Center 1, FL_Loc_+ was a stronger predictor for adverse events than *D*_max_+ and FL_Glo_+, with AUC of 0.88, 0.78, and 0.82, respectively.

**Fig. 6:**
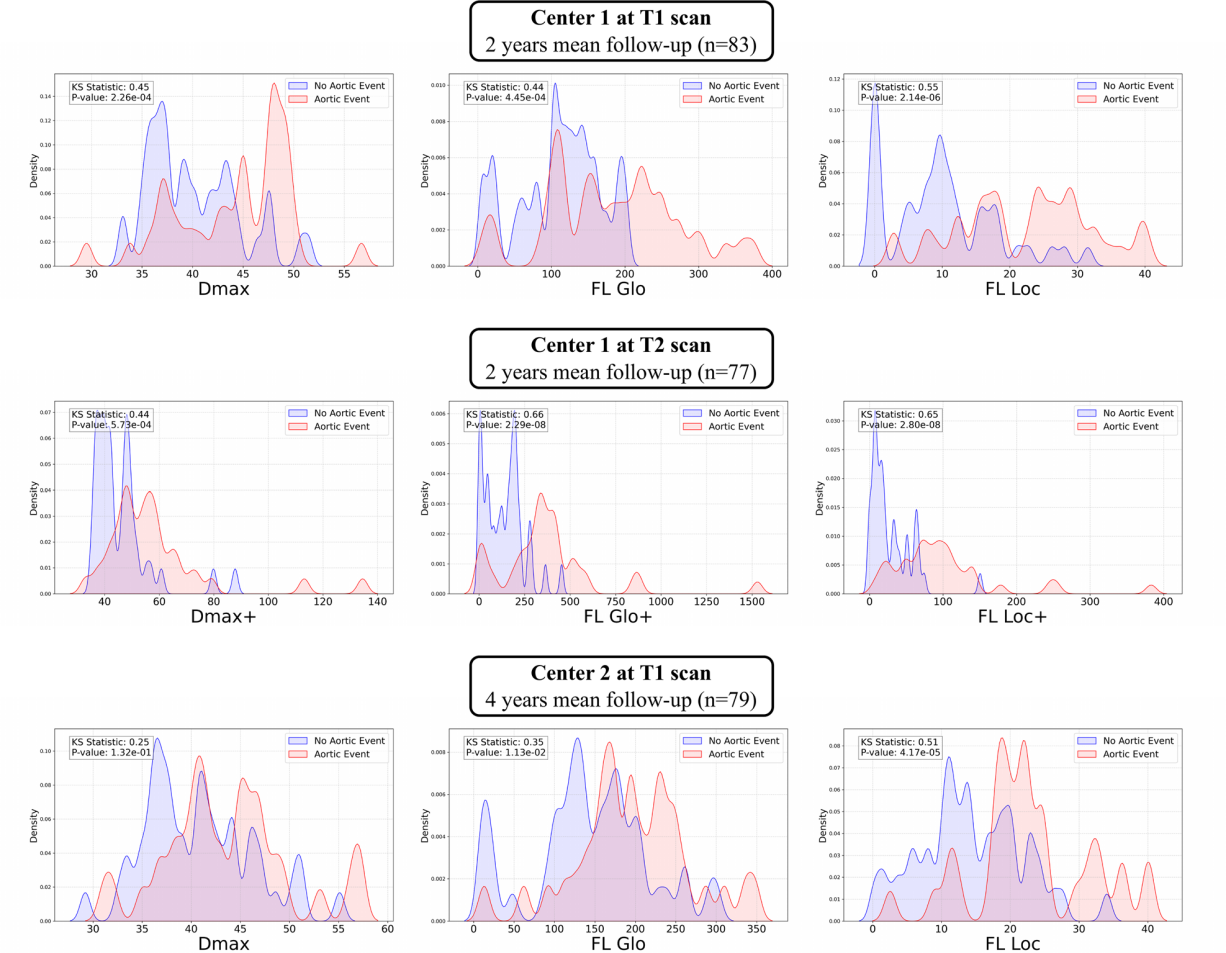
Density plots for each morphological and evolutionary measure on clinical datasets. *D*_max,_ maximal aortic diameter; *FL*_Glo_, global false-lumen volume; *FL*_Loc_, local false-lumen volume. On each subplot, the Kolmogorov–Smirnov (KS) statistic displays the distance between the aortic event distribution and the no aortic event distribution

The predictive performance of automatically obtained aortic measures is presented in Table 5. At baseline (T1), FL_Loc_ demonstrated high sensitivity (Se = 0.87), moderate specificity (Sp = 0.68) with high Predictive negative value (PNV = 0.86) in Center 1 for a threshold of 12.1 mL, which was confirmed in the external validation cohort (Center 2: Se = 0.85, Sp = 0.65, PNV = 0.86, threshold: 17.4 mm). When considering changes between T1 and T2, FL_Loc_ + further improved predictive performance (AUC = 0.88, Sp = 0.95, Sp = 0.70, PPV = 0.93, PNV = 0.86). These findings highlight the added prognostic value of evolution-based markers over static baseline measurements. The study of the effect of FL_Loc_ section length (10-, 15-, 30-, and 50-mm sections) on predictive performance is presented in Supplementary Material Table S2. Regardless of the section length, AUCs of FL_Loc_ were always superior to those of *D*_max_ and FL_Glo_. On density plots, FL_Loc_ provides better discrimination between patients with and without adverse aortic remodeling, particularly in Center 1, compared to *D*_max_ and FL_Glo_ (KS statistics = 0.55, 0.45, and 0.44, respectively, all *p* < 0.001) (Fig. 6).

**Table 5:** Predictive value of automatically obtained aortic measures for adverse aortic remodeling

Center 1 : morphologic measure at T1						
**Measure**	AUC	Threshold*	Sensitivity*	Specificity*	PPV*	PNV*
*D*_max_	0.72	44.7	0.58	0.86	0.79	0.70
FL_Glo_	0.75	202	0.43	0.90	1.0	0.66
FL_Loc_	**0.83**	12.1	0.87	0.68	0.71	0.86
**Center 1: evolution measure (+) between T1 and T2**						
*D*_max_+	0.76	51.2	0.59	0.85	0.79	0.69
FL_Glo+_	0.82	235.7	0.78	0.875	0.85	0.81
FL_Loc+_	**0.88**	66.5	0.70	0.95	0.93	0.78
**Center 2: morphologic measure at T1**						
*D*_max_	0.64	44.5	0.47	0.78	0.61	0.67
FL_Glo_	0.70	160.0	0.76	0.59	0.58	0.77
FL_Loc_	**0.77**	17.4	0.85	0.65	0.64	0.86

+ refers to all evolution measures. (*) thresholds selection based on the Youden index

*FL*_Loc_ local false-lumen volume, *FL*_Glo_ global false-lumen volume, *D*_max_ maximum diameter, *FL*_Glo_/Ao_Length_ global false-lumen volume over vessel length, *FL*_Glo_ /*Ao*_Glo_ global false-lumen over global aortic volume, *PPV* predictive positive value, *PNV* predictive negative value

## Discussion

The main results of the study were as follows: 1) Semantic segmentation driven by a deep learning method provides accurate and reproducible measurements of thoracic aorta diameter and volume for the true-lumen (TL), the CFL, and the thrombus (Th). 2) Local aortic volumes of the false-lumen improve early prediction of RAD adverse aortic remodeling over the maximum aortic diameter and over global false-lumen volume.

Several studies have proposed segmentation of the aorta and aortic dissection. Sieren et al initially proposed a segmentation method of the whole aorta with 0.95 DSC, which is in line with our Ao_Glo_ segmentation [11]. Yu et al tested a 3D convolutional neural network (CNN) for aortic dissection segmentation on 25 patients and reported a DSC of 0.96 for TL and 0.93 for FL [10]. The successive 3D-CNNs of Cao et al were associated with 0.93 DSC in 30 subjects for both TL and FL [9]. Other authors have chosen to straighten the aorta with multiplanar reformation before segmentation. Chen et al specifically advocated the MPR-based method over the others, reporting higher results (0.96 vs. 0.94 DSC for TL) [12]. However, the proximity of the aortic valve may cause the MPR algorithm to truncate the initial part of the aorta, leading to volume errors. Therefore, we used a method that merges unstretched and stretched segmentation. Our reported DSC were similar to previous works, but none of these previous studies compared their method with the interobserver segmentation variability. In their review, Pepe et al highlighted that less common variants of aortic dissection have rarely been considered in these previous works [14]. Except for one recent study [19], thrombus was excluded [10] or confused with false-lumen [13]; variants with stents or extreme aortic tortuosity were all excluded. In contrast, our dataset distinguished thrombus from false-lumen and included various subgroups, reporting no significant difference in performance between them [15, 20] (Supplementary Material Table S4).

Clinical criteria such as male gender, age, and elastic tissue disease are known adverse risk markers of aortic remodeling after dissection [6], as well as a persistent false-lumen after TAAD replacement [3, 21]. However, the most widely used criterion for unfavorable evolution is the maximum aortic diameter on CTA, its evolution, and the size of the entry tear [22]. But it only provides partial information regarding the status of the disease. Measurement of aortic volume has been demonstrated to be a significant risk marker for aortic disease [23]. Initially, Heuts et al highlighted that aortic volumes and length were more accurate than diameter for the occurrence of TAAD [24]. More recently, Gaudry et al showed that global false-lumen volume was an independent predictor of adverse aortic remodeling and superior to maximal diameter [6]. We found similar results with an AUC of 0.76 for FL_Glo_ versus 0.73 for *D*_max_. But manual volume quantification is time-consuming, taking around 30 min, which is not compatible with clinical practice. A fast and reproducible DL method was proposed, thus enabling us to include this measurement in the clinical routine. Another issue is that the global volume measurement lacks specificity. Most cases of adverse aortic remodeling are caused by local aneurysmal evolution, which affects the dissected aorta locally [25]. Global aortic volumes can be underestimated by small local volume changes [26]. Thus, a new radiomarker named FL_Loc_ was automatically computed by the DL model and was the strongest predictive risk marker in our study. This could be due to the local predominance of false-lumen and thrombus, reflecting a weakening of the aortic tissue with hemodynamic changes, which, through collagen degradation, present a risk of aortic remodeling and adverse course [27]. Only focusing on maximal diameter may be a mistake, since a neighboring portion of the aorta may be weakened by hemodynamic pressure. Hence, the local false-lumen was assessed at two different diameter levels instead of one, with a significant performance improvement (see Supplementary Material C.4).

This concept aligns with the growing evidence that local rather than global morphological changes drive adverse remodeling. In line with this, Fleischmann et al, in a multi-center study, recently confirmed the major prognostic role of maximal aortic diameter, while other morphological features such as false-lumen circumferential angle, outflow, or intercostal arteries were not predictive [28]. Their model, however, did not include volumetric parameters. Our findings, therefore, complement theirs, showing that integrating local false-lumen volume (FL_Loc_), a physio-pathologically meaningful marker, may further improve risk prediction beyond diameter alone.

Gaudry et al proposed evolution markers at 3 months and one year, but these proved less effective than the values at T1. Our results show that time-normalized evolution markers are the most discriminant parameters since they extrapolate the observed T2-T1 difference at a fixed date (one year after T1), accounting for the variability in days between CTA. This can provide an estimation of the future aortic measure one year after T1.

Our study has some limitations. Firstly, our segmentation dataset did not fully capture the complete spectrum of aortic dissections; in particular, multichannel aortic dissections were absent. This limitation was reduced by including all patients with RAD or TBAD without anatomical or phenotypical exclusions and by using patients from two different centers. Secondly, these experiments need to be replicated on a prospective longitudinal cohort. The clinical set used to investigate risk markers was relatively small, and the average follow-up was short, yet half of the patients had an adverse outcome.

## Conclusion

A unique multi-space DL model segmenting all the aortic dissection components, including thrombus, was developed, recording automatic measurements of aortic length, diameters, and volumes. The local volume of the false-lumen around the maximum diameter is a significant prognostic factor for adverse aortic remodeling in patients with RAD, and its evolution is even more predictive. Larger clinical studies to assess the potential impact of such a technique in clinical practice will be mandatory.

## Supplementary information

Supplementary information

## Abbreviations

Ao_Glo_: Global aortic volume
Ao_length_: Aortic length
Ao_Loc_: Local aortic volume
CFL: Circulating false-lumen
CTA: Computed tomography angiography
*D*_max_: Maximal aortic diameter
FL_Glo_: Global false-lumen volume
FL_Loc_: Local false-lumen volume
RAD: Residual aortic dissection
TAAD: Type A aortic dissection
TBAD: Type B aortic dissection
Th: Thrombus
TL: True-lumen

## References

[1] Criado FJ (2011) Aortic dissection: a 250-year perspective. Tex Heart Inst J 38:694–700 22199439 PMC3233335

[2] Isselbacher EM, Preventza O, Hamilton Black J et al (2022) 2022 ACC/AHA Guideline for the Diagnosis and Management of Aortic Disease: A Report of the American Heart Association/American College of Cardiology Joint Committee on Clinical Practice Guidelines. Circulation. 146. 10.1161/CIR.0000000000001106 10.1161/CIR.0000000000001106 PMC9876736 36322642

[3] Evangelista A, Salas A, Ribera A et al (2012) Long-term outcome of aortic dissection with patent false lumen: predictive role of entry tear size and location. Circulation 125:3133–3141. 10.1161/CIRCULATIONAHA.111.090266 22615344 10.1161/CIRCULATIONAHA.111.090266

[4] Zierer A, Voeller RK, Hill KE, Kouchoukos NT, Damiano RJ, Moon MR (2007) Aortic enlargement and late reoperation after repair of acute type A aortic dissection. Ann Thorac Surg 84:479–487. 10.1016/j.athoracsur.2007.03.084 17643619 10.1016/j.athoracsur.2007.03.084

[5] Adriaans BP, Wildberger JE, Westenberg JJM, Lamb HJ, Schalla S (2019) Predictive imaging for thoracic aortic dissection and rupture: moving beyond diameters. Eur Radiol 29:6396–6404. 10.1007/s00330-019-06320-7 31278573 10.1007/s00330-019-06320-7 PMC6828629

[6] Gaudry M, Guivier-Curien C, Blanchard A et al (2022) Volume analysis to predict the long-term evolution of residual aortic dissection after type A repair. J Cardiovasc Dev Dis 9:349. 10.3390/jcdd9100349 36286301 10.3390/jcdd9100349 PMC9604488

[7] Sobocinski J, Lombardi JV, Dias NV et al (2016) Volume analysis of true and false lumens in acute complicated type B aortic dissections after thoracic endovascular aortic repair with stent grafts alone or with a composite device design. J Vasc Surg 63:1216–1224. 10.1016/j.jvs.2015.11.037 26806523 10.1016/j.jvs.2015.11.037

[8] Higashigaito K, Sailer AM, Van Kuijk SMJ et al (2021) Aortic growth and development of partial false lumen thrombosis are associated with late adverse events in type B aortic dissection. J Thorac Cardiovasc Surg 161:1184–1190.e2. 10.1016/j.jtcvs.2019.10.074 31839226 10.1016/j.jtcvs.2019.10.074 PMC10552621

[9] Cao L, Shi R, Ge Y et al (2019) Fully automatic segmentation of type B aortic dissection from CTA images enabled by deep learning. Eur J Radiol 121: 108713. 10.1016/j.ejrad.2019.108713 31683252 10.1016/j.ejrad.2019.108713

[10] Yu Y, Gao Y, Wei J et al (2021) A three-dimensional deep convolutional neural network for automatic segmentation and diameter measurement of type B aortic dissection. Korean J Radiol 22:168–178. 10.3348/kjr.2020.0313 33236538 10.3348/kjr.2020.0313 PMC7817629

[11] Sieren MM, Widmann C, Weiss N et al (2022) Automated segmentation and quantification of the healthy and diseased aorta in CT angiographies using a dedicated deep learning approach. Eur Radiol 32:690–701. 10.1007/s00330-021-08130-2 34170365 10.1007/s00330-021-08130-2

[12] Chen D, Zhang X, Mei Y et al (2021) Multi-stage learning for segmentation of aortic dissections using a prior aortic anatomy simplification. Med Image Anal 69: 101931. 10.1016/j.media.2020.101931 33618153 10.1016/j.media.2020.101931

[13] Hahn LD, Mistelbauer G, Higashigaito K et al (2020) CT-based true- and false-lumen segmentation in type B aortic dissection using machine learning. Radio Cardiothorac Imaging 2:e190179. 10.1148/ryct.2020190179 10.1148/ryct.2020190179 PMC7977949 33778582

[14] Pepe A, Li J, Rolf-Pissarczyk M et al (2020) Detection, segmentation, simulation and visualization of aortic dissections: a review. Med Image Anal 65: 101773. 10.1016/j.media.2020.101773 32738647 10.1016/j.media.2020.101773

[15] Varoquaux G, Cheplygina V (2022) Machine learning for medical imaging: methodological failures and recommendations for the future. NPJ Digit Med 5:48. 10.1038/s41746-022-00592-y 35413988 10.1038/s41746-022-00592-y PMC9005663

[16] Gaudry M, Porto A, Guivier-Curien C et al (2021) Results of a prospective follow-up study after type A aortic dissection repair: a high rate of distal aneurysmal evolution and reinterventions. Eur J Cardiothorac Surg 61:152–159. 10.1093/ejcts/ezab317 34355742 10.1093/ejcts/ezab317

[17] Fedorov A, Beichel R, Kalpathy-Cramer J et al (2012) 3D Slicer as an image computing platform for the quantitative imaging network. Magn Reson Imaging 30:1323–1341. 10.1016/j.mri.2012.05.001 22770690 10.1016/j.mri.2012.05.001 PMC3466397

[18] Belvroy VM, de Beaufort HWL, van Herwaarden JA, Bismuth J, Moll FL, Trimarchi S (2019) Tortuosity of the descending thoracic aorta: normal values by age. PLoS One 14:e0215549. 10.1371/journal.pone.0215549 31013307 10.1371/journal.pone.0215549 PMC6478292

[19] Feng H, Fu Z, Wang Y, Zhang P, Lai H, Zhao J (2023) Automatic segmentation of thrombosed aortic dissection in post-operative CT-angiography images. Med Phys 50:3538–3548. 10.1002/mp.16169 36542417 10.1002/mp.16169

[20] Petersen E, Holm S, Ganz M, Feragen A (2023) The path toward equal performance in medical machine learning. Patterns 4: 100790. 10.1016/j.patter.2023.100790 37521051 10.1016/j.patter.2023.100790 PMC10382979

[21] Kimura N, Itoh S, Yuri K et al (2015) Reoperation for enlargement of the distal aorta after initial surgery for acute type A aortic dissection. J Thorac Cardiovasc Surg 149:S91–98.e1. 10.1016/j.jtcvs.2014.08.008 25224548 10.1016/j.jtcvs.2014.08.008

[22] Leontyev S, Haag F, Davierwala PM et al (2017) Postoperative changes in the distal residual aorta after surgery for acute type A aortic dissection: impact of false lumen patency and size of descending aorta. Thorac Cardiovasc Surg 65:90–98. 10.1055/s-0036-1571813 27111499 10.1055/s-0036-1571813

[23] Sigman MM, Palmer OP, Ham SW, Cunningham M, Weaver FA (2014) Aortic morphologic findings after thoracic endovascular aortic repair for type B aortic dissection. JAMA Surg 149:977–983. 10.1001/jamasurg.2014.1327 25075710 10.1001/jamasurg.2014.1327

[24] Heuts S, Adriaans BP, Rylski B et al (2020) Evaluating the diagnostic accuracy of maximal aortic diameter, length and volume for prediction of aortic dissection. Heart 106:892–897. 10.1136/heartjnl-2019-316251 32152004 10.1136/heartjnl-2019-316251

[25] Zhu Y, Xu XY, Rosendahl U, Pepper J, Mirsadraee S (2022) Prediction of aortic dilatation in surgically repaired type A dissection: A longitudinal study using computational fluid dynamics. JTCVS Open 9:11–27. 10.1016/j.xjon.2022.01.019 36003481 10.1016/j.xjon.2022.01.019 PMC9390758

[26] Qin YL, Deng G, Li TX, Jing RW, Teng GJ (2012) Risk factors of incomplete thrombosis in the false lumen after endovascular treatment of extensive acute type B aortic dissection. J Vasc Surg 56:1232–1238. 10.1016/j.jvs.2012.04.019 22795522 10.1016/j.jvs.2012.04.019

[27] Zimmermann J, Bäumler K, Loecher M et al (2023) Hemodynamic effects of entry and exit tear size in aortic dissection evaluated with in vitro magnetic resonance imaging and fluid-structure interaction simulation. Sci Rep 13:22557 10.1038/s41598-023-49942-0 PMC10728172 38110526

[28] Fleischmann D, Mastrodicasa D, Willemink MJ et al (2025) Predicting late adverse events in uncomplicated Stanford type B aortic dissection: results from the ROADMAP validation study. Circ Cardiovasc Imaging 18:e016766. 10.1161/CIRCIMAGING.124.016766 39965039 10.1161/CIRCIMAGING.124.016766 PMC11839160

