# Supplementary material for: Deep learning-driven false-lumen volumes predict adverse remodeling better than diameter in patients with residual aortic dissection on CT
Source: Eur Radiol. 2025 Nov 7;36(4):3257–70. doi: 10.1007/s00330-025-12116-9 (PMC13035661; doi:10.1007/s00330-025-12116-9)

Deep learning-driven false-lumen volumes predict adverse remodeling better than diameter in patients with residual aortic dissection on CT

ELECTRONIC SUPPLEMENTARY MATERIAL

Supplementary A: CTA data

A.1. CTA acquisition protocol in Center 1.

All total aortic CTA examinations were performed on a 128-slice CT scanner (Revolution EVO, General Electric Healthcare) with high-pitch helical non-ECG-gated acquisition and craniocaudal acquisition from the base of skull to the femoral artery bifurcations. The imaging parameters were: 100 kV for patients < 80kg to 120kV for patients >80kg. Auto mAs slice thickness:0.625mm, interval:0.625mm. rotation time:0.4s, pitch:0.984. Injection protocol was a dose of 1mL/kg of iodine contrast medium at a flow-rate of 5mL/s and 40mL saline chaser. Acquisition was triggered by bolus tracking in the descending aorta. A delayed acquisition 50 seconds after injection was performed.

A.2. CTA acquisition protocol in Center 2.

All total aortic CTA examinations were performed on a third-generation DSCT (SOMATOM Force, Siemens) with a high-pitch helical non-gated acquisition and craniocaudal acquisition from the base of skull to the femoral artery bifurcations. The imaging parameters were: automatically selected tube voltage, collimation 192 mm x0.6mm, pitch 3.2, slice thickness: 0.75mm. Injection protocol was a biphasic injection with 70mL of contrast at a rate of 5.0 mL/s, followed by 20mL of contrast at 3.5 mL/s and a saline flush of 25ml. Acquisition was triggered by bolus tracking of 100UH in the descending aorta. A delayed acquisition 70 seconds after injection was performed.

Supplementary B: model development

B.2. Automatic segmentation pipeline development.

The segmentation pipeline (illustrated in Supplementary Figure 1) starts with pre-processing and 1mm isotropic resampling of the CTA, forwarded to a 3D-UNet segmenting TL, CFL and Th. The aortic centerline was extracted from the segmentation to create a multi-planar reformatted (MPR) image, in which the dissection components are segmented a second time by another 3D-UNet in order to obtain a robust diameter measurement. Finally, the CTA and MPR segmentations are fused by adding their softmax outputs after retro-projection into original space, and clinical measurements are derived: Dmax (on MPR), volumes and FLLoc. The model can be shared with colleagues subject to a signed agreement.

B.3. Neural networks implementation details

Parameterized with nnUNet, both networks are U-Net networks with 5 encoder blocks and a central bottleneck, with an input size of 96x96x256 and 64x64x512 respectively. Both networks used LeakyReLU and instance normalisation. The cost function was the dice loss summed with categorical cross entropy. Deep supervision was used in the last four scales (from 0.125 to 1) of the decoder; the Nesterov SGD optimiser with a momentum of 0.99; an initial learning rate of 0.01 with a decay weight of 3e-05. Crops, rotations, gamma corrections were applied randomly during the 1000 training epochs of each network. The training time was approximately one week per network on our computer with the following characteristics Intel Xeon Silver 4216 2.1GHz dual processor, RAM = 96GB, 2 Nvidia Quadro RTX5000 GPUs, 16GB.

B.4. Risk parameters: evolution criteria formula

The most elementary approach of an evolution criterion C is the gross difference between the value at T2 and T1:

$$C_{T2} - C_{T1}$$

However it doesn't account for the variable number of days $(T2 - T1)$ between the two scanners. We therefore use a coefficient which acts as a time normalizer:

$$\frac{C_{T2} - C_{T1}}{t_{coef}}$$

$$t_{coef} = \frac{T2 - T1}{365}$$

There is another important aspect to consider when assessing evolutions. Suppose we have two patients A and B with RAD: at T1 scan, A's maximum diameter is 35mm, B's is 45mm.

At T2, both A and B's maximum diameter had gained 5mm. It's obvious the 5mm gain has more impact for B than for A, therefore our evolution criterion should reflect this difference.

To do so, we simply add our time-normalized difference to the initial T1 value:

$$C_{T1} + \frac{C_{T2} - C_{T1}}{t_{coef}}$$

Finally, sometimes only looking at the T2 value can be more informative about the future:

$$C{+} = \max\left(C_{T2}, C_{T1} + \frac{C_{T2} - C_{T1}}{t_{coef}}\right)$$

This supplementary provides experimental comparison between $C+$ and the gross approach $C_{T2} - C_{T1}$.

The T1 and T2 CTAs might include a different proportion of the entire aorta. Computing the volume evolutions with the gross quantifications in such a case would

result in biased values. We therefore equalize the two segmentations by cutting the longest aorta, downstream, so that its length equates the other vessel length.

B.5. Detailed procedure to compute the local false volume criterion

At T1, two candidate levels are identified for future aneurysm development: the maximum diameter position (D1) and the second largest diameter position (D2), and calculate the local (FL_{Loc}) of false lumen volume. More specifically, as shown in manuscript Figure 3:

1. The diameters, measured on the stretched aorta, are sorted in descending order, to identify the two maximum diameter positions on the aortic centerline, D1 and D2.
2. Using the stored orthogonal planes of the MPR algorithm and the centerline, two portions, P_{D1} and P_{D2}, are cut around the two candidate levels.
3. In each portion, the local volumes are measured as weighted sums of the false lumen and thrombus volumes:

$$FL_{D1} = CFL(P_{D1}) + \alpha Th(P_{D1}) \text{ and } FL_{D2} = CFL(P_{D2}) + \alpha Th(P_{D2})$$

4. Finally, the largest sum is kept:

$$FL_{Loc} = \max(FL_{D1}, FL_{D2})$$

Grid search fixed α to 0.75.

Compared to other evolution criteria, FL_{Loc}+ has one component for each diameter position:

$$FL_{Loc} += A + B$$

where $A = \max\left(FL_{D1}^{T1}, FL_{D1}^{T2}, \widehat{FL}_{D1}^{T1+1year}\right)$ and $B = \max\left(FL_{D2}^{T1}, FL_{D2}^{T2}, \widehat{FL}_{D2}^{T1+1year}\right)$,

with $\widehat{FL}_{D}^{T1+1year} = FL_{D}^{T1} + \frac{FL_{D}^{T1} - FL_{D}^{T0}}{t_{coef}}$.

Supplementary C: Additional experiments

C.1. Additional experiment 1: T2+ evolution approach versus T2-T1 gross difference

Supplementary Table S1 compares the AUC scores with 95% intervals between our T2+ evolution approach and the T2-T1 gross difference. The T2+ approach outperformed the gross differences for the 5 markers. In particular, FL_{Loc} increases from 0.73 to 0.86 (+), FL_{Glo} /Ao_{Glo} from 0.69 to 0.81. The smallest AUC difference was observed for FL_{Glo}: 0.78 versus 0.82 (+).

C.2. Studying effect of choosing different aortic portion length in FL_{Loc}

Supplementary Table S2 contains the AUCs associated to different range length (10mm, 15mm, 30mm, 50mm, 75mm).

C.3. Additional experiment 2: Should we combine the criteria into one machine learning model to predict the RAD adverse course?

In this experiment, we investigated the prediction accuracy of random forests with one criterion or with several criteria as input. Before, we turned each parameter into a binary variable applying an optimal threshold value given by CART algorithm. Prediction accuracies are given in Supplementary Table S3.

At T1, combining the criteria as input resulted in a superior accuracy: 81% (FL_{Loc} and FL_{Glo} /Ao_{Length} and FL_{Glo} /Ao_{Glo}) versus 79.5% for the best single input (FL_{Loc}). At T2 the gap was even more significant in favor or criteria combination: 91% (FL_{Loc}+ and FL_{Glo} /Ao_{Length}+ and FL_{Glo} /Ao_{Glo}+ and DMax+) versus 83.5% for FL_{Loc}+ alone.

C.4. Additional experiment 3: Should we quantify FL_{Loc} around the two largest diameters or only around the maximum diameter?

Quantifying FL_{Loc} with 15mm range only around the maximum diameter position was associated with an AUC of 0.72 against 0.82 around the two largest diameter positions. Random forest predictive accuracy sets at 69.8% versus 79.5%, respectively. Likelihood ratio test reveals that adding the local volume marker with second level to a baseline regression model with one level, result in a significant performance gain (p<0.001).

For FL_{Loc}+, the AUC were of 0.84 against 0.86 and RF accuracies of 80.8% against 83.5%, with one or two levels respectively. Likelihood ratio *p*-value was also significant (p=0.009) when adding the latter over the former in a regression model.

C.5. Additional experiment 4: Segmentation performance on rare dissection variants.

Supplementary Table S4 displays the segmentation performance over the different variants. The mean DSC for the thrombus (0.94, 0.93 and 0.87 DSC for VC, FC and TH respectively) and tortuous (0.93, 0.93 and 0.86) groups were almost identical to those of the full testset (0.93, 0.93 and 0.87). The presence of a metal stent slightly disturbed this balance, with the true lumen achieving a higher DSC (0.95 vs. 0.93), but the false lumen a lower one (0.90 vs. 0.93), as did the thrombus (0.85 vs. 0.87), however none of those differences were significant.

Supplementary Tables

Supplementary Table S1. Comparison of our evolution criteria with raw difference in terms of AUC values with 95% confidence intervals.

Note.— FL_{Loc}: local false lumen volume; FL_{Glo}: global false lumen volume; Dmax: maximum diameter; FL_{Glo} /Ao_{Length} : global false lumen volume over vessel length; FL_{Glo} /Ao_{Glo} : global false lumen over global aortic volume. + refers to all evolution measures.

	FL_{Loc}	Dmax	FL_{Glo}	FL_{Glo} /Ao_{Glo}	FL_{Glo} /Ao_{Length}
AUC Gross T2-T1 difference	0.73 [0.60-0.85]	0.69 [0.55-0.81]	0.78 [0.67-0.89]	0.69 [0.57-0.81]	0.78 [0.67-0.89]
AUC T2+ approach	**0.88** [0.79-0.95]	**0.79** [0.68-0.88]	**0.82** [0.72-0.92]	**0.81** [0.70-0.92]	**0.84** [0.73-0.93]

Supplementary Table S2. AUC values to compare choices of ranges in local false lumen volume.

Local volume range	AUC T1 (Center 1)	AUC T2+ (Center 1)	AUC T1 (Center 2)
10mm	0.79 [0.69, 0.89]	0.86 [0.77, 0.94]	0.76 [0.66, 0.86]
15mm	0.82 [0.72, 0.91]	0.86 [0.77, 0.94]	0.76 [0.64, 0.85]
30mm	0.83 [0.73, 0.92]	**0.88 [0.79, 0.95]**	**0.77 [0.67, 0.87]**
50mm	**0.84 [0.75, 0.92]**	0.87 [0.78, 0.94]	**0.77 [0.67, 0.87]**
75mm	0.84 [0.74, 0.92]	0.87 [0.78, 0.94]	x

Supplementary Table S3. Prediction accuracies for random forest with single or multiple input.

Note.— FL_{Loc}: local false lumen volume; FL_{Glo}: global false lumen volume; Dmax: maximum diameter; FL_{Glo} /Ao_{Length} : global false lumen volume over vessel length; FL_{Glo} /Ao_{Glo} : global false lumen over global aortic volume. + refers to all evolution measures.

				T1		
Input	FL_{Loc}	Dmax	FL_{Glo}	FL_{Glo} /Ao_{Glo}	FL_{Glo} /Ao_{Length}	FL_{Loc} and FL_{Glo} /Ao_{Length} and FL_{Glo} /Ao_{Glo}
Accuracy	79.5%	72.5%	72.5%	74.5%	74.5%	**82%**
				**T2+**		
Input	FL_{Loc}+	Dmax+	FL_{Glo}+	FL_{Glo} /Ao_{Glo}+	FL_{Glo} /Ao_{Length}+	FL_{Loc}+ and FL_{Glo} /Ao_{Length}+ and FL_{Glo} /Ao_{Glo}+ and Dmax+
Accuracy	83.5%	77%	82%	83%	83%	**91%**

Supplementary Table S4. Segmentation performances on dissection variant subgroups: Mean DSC.

Note.— DSC: dice similarity coefficient.

Class	True Lumen	False Lumen	Thrombus
Thrombus (n=15)	0.94 ± 0.02	0.93 ± 0.04	0.87 ± 0.06
Ext.Tort. (n=5)	0.93 ± 0.02	0.93 ± 0.05	0.86 ± 0.07
Stent Graft (n=7)	0.95 ± 0.01	0.90 ± 0.05	0.85 ± 0.04
All (n=30)	0.93 ± 0.03	0.93 ± 0.04	0.87 ± 0.06

Supplementary Figures

Supplementary Figure S1. Illustration of the segmentation pipeline. M_{ori} is a 3D nnU-Net working in the original angiographic space; M_{mpr} a 3D nnU-Net segmenting in the multi-planar reformatted (MPR) space; M_{mix} is the resulting fusion of the two predictions.

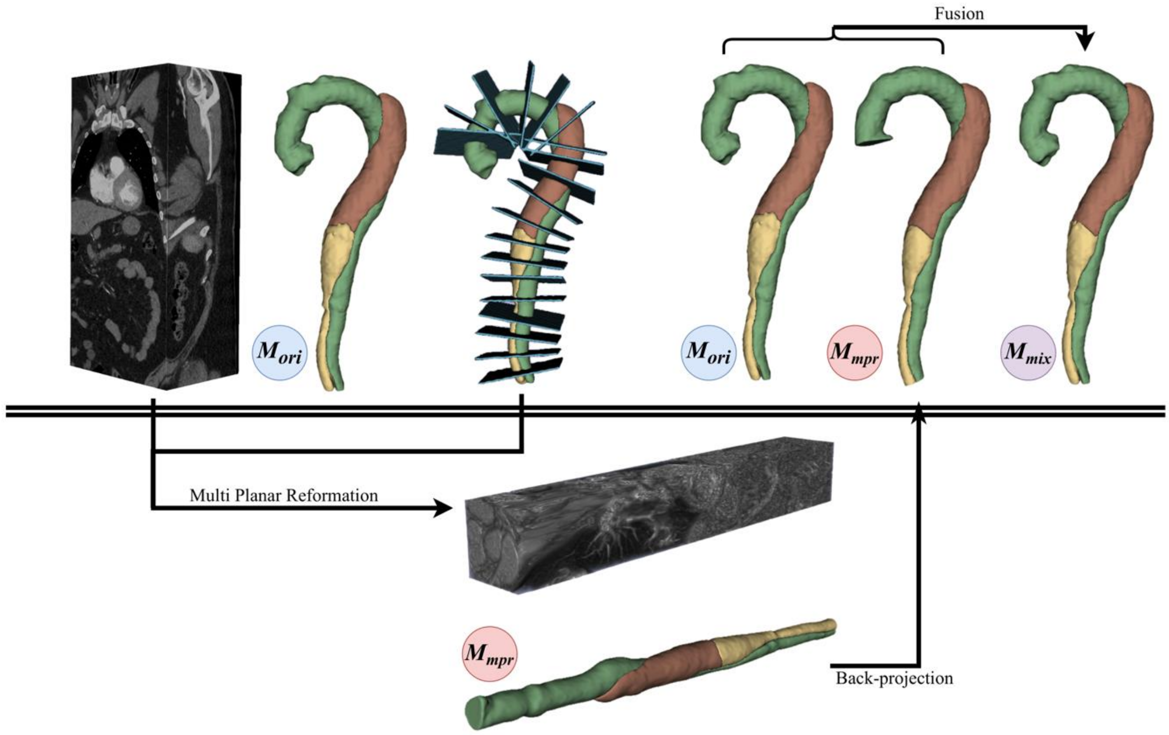

Supplementary Figure S2. **Case example : an automated and a manual segmentation of a thrombosed aortic dissection.**
Note.— Left: Sagital view; right: two axial views.

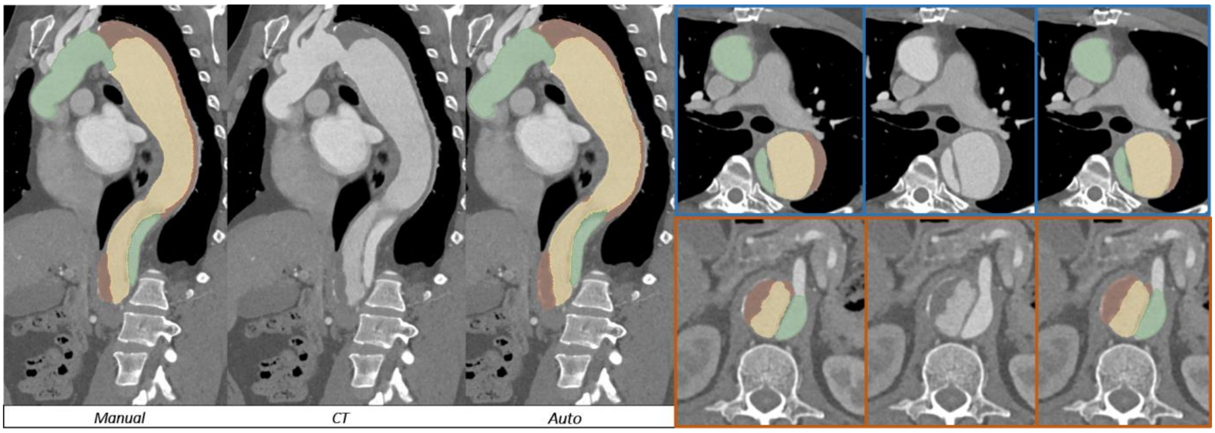

Supplement: Supplementary file 1 — Supplementary information [file 330_2025_12116_MOESM1_ESM.pdf]
